# Rhinoviruses and Respiratory Enteroviruses: Not as Simple as *ABC*

**DOI:** 10.3390/v8010016

**Published:** 2016-01-11

**Authors:** Léna Royston, Caroline Tapparel

**Affiliations:** 1 University of Geneva Faculty of Medicine, 1 Rue Michel-Servet, 1205 Geneva, Switzerland; caroline.tapparel@hcuge.ch; 2 Laboratory of Virology, Division of Infectious Diseases, University of Geneva Hospitals, 4 Rue Gabrielle Perret-Gentil, 1211 Geneva 14, Switzerland

**Keywords:** rhinovirus, enterovirus, respiratory virus, common cold, pathogenesis, evolution

## Abstract

Rhinoviruses (RVs) and respiratory enteroviruses (EVs) are leading causes of upper respiratory tract infections and among the most frequent infectious agents in humans worldwide. Both are classified in the *Enterovirus* genus within the *Picornaviridae* family and they have been assigned to seven distinct species, RV-A, B, C and EV-A, B, C, D. As viral infections of public health significance, they represent an important financial burden on health systems worldwide. However, the lack of efficient antiviral treatment or vaccines against these highly prevalent pathogens prevents an effective management of RV-related diseases. Current advances in molecular diagnostic techniques have revealed the presence of RV in the lower respiratory tract and its role in lower airway diseases is increasingly reported. In addition to an established etiological role in the common cold, these viruses demonstrate an unexpected capacity to spread to other body sites under certain conditions. Some of these viruses have received particular attention recently, such as EV-D68 that caused a large outbreak of respiratory illness in 2014, respiratory EVs from species C, or viruses within the newly-discovered RV-C species. This review provides an update of the latest findings on clinical and fundamental aspects of RV and respiratory EV, including a summary of basic knowledge of their biology.

## 1. Introduction

Rhinoviruses (RVs) are responsible for more than one-half of upper respiratory tract infections (URTI) and they are considered to be among the most frequent infectious agents in humans worldwide [1]. Most cases of RV infections are benign, self-limited cold-like illnesses. However, these viruses have been also identified as the causal agent of severe pneumonia in the elderly and immunocompromised patients, as well as exacerbations of chronic obstructive pulmonary disease and asthma. At present, no efficient antiviral treatment, vaccines, or other preventive measures exist against these particularly frequent pathogens (apart from poliovirus). In addition to the significant associated clinical morbidities, the economic impact of RV-related infections is of great concern [2]. Viral URTI are highly expensive for society, both directly (healthcare resource use) and indirectly (productivity loss), which emphasizes the importance of finding an appropriate preventive treatment.

RVs belong to the *Enterovirus* (EV) genus within the *Picornaviridae* family. The members of this genus are divided into seven human species, three RV species (RV-A to RV-C) and four EV (non-RV EV) species (EV-A to EV-D). Although closely related at a genetic level, these viruses have remarkably different phenotypic characteristics. The tropism of RVs is restricted to upper respiratory airways, except in some rare cases of disseminated disease, whereas EVs can infect a wide range of different cells and cause very diverse clinical syndromes [3]. Diseases due to non-RV EVs range from febrile illnesses to myopericarditis, paralysis or encephalitis, with a significant number of complications and deaths. EVs are notably the most frequent cause of viral meningitis [4]. However, some types of EVs are only found in the respiratory tract and cause RV-like symptoms, especially EVs from species C and D (Table 1), and are consequently named respiratory EVs. Some have been shown to share characteristic traits of RVs, including instability at low pH (<5–6) or high temperatures (>34 °C) [5].

This review provides an overview of the latest findings on the clinical and fundamental aspects of RVs and respiratory EVs and briefly summarizes current knowledge of RV and EV biology.

**Table 1:** Non-Rhinovirus (RV) Enteroviruses (EVs) associated with respiratory diseases.

Species	Types of Viruses Detected Occasionally in Respiratory Samples	Types of Viruses Detected Predominantly or Exclusively in Respiratory Samples
EV-A	CV-A10, CV-A16, EV-A71	–
EV-B	CV-A9, CV-B1, CV-B2, CV-B3, CV-B4, CV-B5, CV-B6, E-1, E-2, E-3, E-4, E-5, E-6, E-7, E-9, E-11, E-12, E-13, E-14, E-15, E-16, E-17, E-18, E-19, E-20, E-21, E-25, E-29, E-30	–
EV-C	CV-A24, PV-3	EV-C104 [6], EV-C105 [7], EV-C109 [8], EV-C117 [9], EV-C118 [10], CV-A21
EV-D	_–_	EV-D68

Adapted from [3].

## 2. Overview of Rhinovirus Biology

### 2.1. Brief Overview of Basic Virology

#### 2.1.1. Genome and Structure

RVs and EVs are small, non-enveloped, positive-stranded RNA viruses with a genome of about 7.2 to 7.5 kb packed in a 30 nm icosahedric capsid. This capsid is composed of the assembly of 12 pentamers of 5 protomers, consisting of the four capsid proteins VP1, VP2, VP3 and VP4. VP1 is located at the external side and is the major target of the immune response [11], even if VP2 and VP3 contribute to the antigenicity. VP4 is localized on the internal surface of the capsid and interacts with the genome.

**Figure 1:**
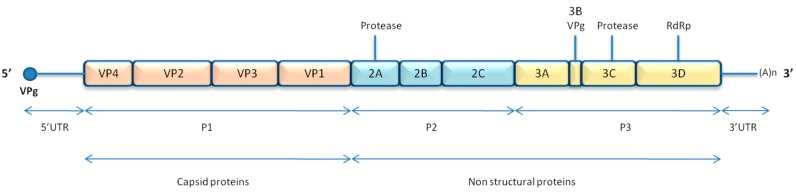
Enterovirus genome organisation.

An internal ribosomal entry site (IRES) is located in the 5′untranslated region of the genome (5′UTR) and is necessary for translation. This translation gives rise to a precursor polyprotein, which is cleaved by viral proteases in 11 mature proteins. The single open reading frame is divided into three regions: the first region of the genome, P1, encodes for capsid proteins (VP1 to VP4), whereas the next regions, P2 and P3, give rise to non-structural proteins (2A to 2C and 3A to 3D) (Figure 1). Regarding structural elements, a cloverleaf structure is located close to the IRES in the 5′-UTR and a small stem loop in the 3′-UTR. Another stem loop structure, the *cis*-acting replication element (*cre*), is positioned at different places throughout the polyprotein coding region, depending on the viral species. A small viral protein, VPg, is covalently bound to the 5′ end of the genome, but seems to be cleaved from the genomic RNA early in the replication cycle. This protein is implicated in priming viral genome for replication, but recent studies have reported that its presence does not affect translation or replication [12,13]. Thus, it is unclear why this peptide is cleaved from genomic RNA shortly after its release in the cytoplasm, and it has been speculated that VPg unlinking may be necessary for proper encapsidation of newly synthesized genomic RNAs in particles as only VPg-containing viral RNA is found in virions [13].

#### 2.1.2. Replication Cycle

Virus entry to the cell depends on the cell surface molecule used as receptor, as well as putative attachment receptors that vary between the different EV and RV types. Viral uptake can be mediated either by clathrin-dependent or -independent endocytosis or via macropinocytosis, depending on the virus and the host cell type [14]. Virions then undergo a conformational change triggered by the drop in pH or by receptor binding and leads to uncovering of the hydrophobic domains, which results in pore-mediated release of the genome in the cytoplasm. A cap-independent IRES-mediated polyprotein synthesis is then mediated by the host cell ribosomal machinery. The obtained viral polyprotein precursor of approximately 2000 amino acids is cleaved by the viral proteases into different viral proteins. The genome is then replicated in complexes in association with cytoplasmic membranes [15]. For more details, the EV replication cycle was recently reviewed [16].

### 2.2. Pathogenesis and Associated Diseases

#### 2.2.1. Transmission

The transmission of viral particles between humans occurs mainly via direct contact or through a fomite, typically with inoculation into the eye or nose from the fingertip. RVs are able to survive on hands for several hours, which allows an easy human-to-human transmission through this route in the absence of adequate hand hygiene, particularly in the presence of high viral loads [17]. Transmission by large particle aerosols has also been documented, but is presumably less efficient [18].

#### 2.2.2. Site of Infection and Pathogenesis

Inoculation of RV happens either directly on the nasal mucosa or via the eye conjunctiva where it is transported via the lacrymal duct to the nasal cavity, and then on to the nasopharynx. The airway epithelium is the primary site of infection of RV and it was shown in studies of both natural and experimentally-induced cold that viral RNA cannot be detected in the subepithelial layer, only in epithelial cells. Most RV-A and -B utilize intercellular adhesion molecule (ICAM)-1 as cell entry receptor (classified as the major receptor group) and the others alternatively bind low density lipoprotein receptor (LDL-R) (minor receptor group) (Table 2), whereas RV-C utilizes a different receptor molecule (see Update section). These receptors are expressed by ciliated and non-ciliated airway cells. *In situ* hybridization experiments reported that RVs replicate in a small proportion of these cells in the nasopharynx and nasal epithelium of experimentally-infected humans [19]. ICAM-1 expression is limited in cells, but RV infection increases surface ICAM-1 expression via nuclear factor (NF)-κB p65-mediated transcriptional up-regulation [20]. This up-regulation was observed *in vitro* in normal primary human bronchial epithelial cells (HBECs), but also *in vivo* when infecting experimentally healthy human volunteers [21]. Unlike other respiratory viruses, RV by itself does not cause airway epithelial cell destruction and a visible cytopathic effect [22]. Yet, even if the epithelium morphology remains intact, RV compromises the epithelial barrier function by dissociating zona occludens 1 from the tight junction complex by stimulating reactive oxygen species (ROS) generation during viral replication [23]. This disruption of the barrier function increases pathogen (including bacteria) translocation across the polarized airway epithelial cells (AECs), which can lead to complicated disease [24].

**Table 2:** Cellular receptors for RVs and non-RV respiratory EVs.

NON-RV Respiratory Enteroviruses			Rhinoviruses		
Species	Genotype	Receptor	Species	Genotype	Receptor
EV-A	CV-A10	unknown	RV-A	A7, A8, A9, A10, A11, A12, A13, A15, A16, A18, A19, A20, A21, A22, A24, A27, A28, A32, A33, A34, A36, A38, A39, A40, A41, A43, A45, A46, A50, A51, A53, A54, A55, A56, A57, A58, A59, A60, A61, A63, A64, A65, A66, A67, A68, A71, A73, A74, A75, A76, A77, A78, A80, A81, A82, A85, A88, A89, A90, A94, A96, A100, A101, A102, A103, A104, A105, A106, A107, A108, A109	ICAM-1 [25]
	CV-A16	SCARB2 [26], PSGL-1 [27]			
	EV-A71	SCARB2 [26], PSGL-1 [27] *			
EV-B	CV-A9	αV integrins [28,29,30]			
	CV-B1	CAR [31,32], DAF [33]			
	CV-B2	CAR [31,32]			
	CV-B3	CAR [31,32], DAF [33]			
	CV-B4	CAR [31,32]			
	CV-B5	CAR [31,32], DAF [33]			
	CV-B6	CAR [31,32]			
	E-1	α2β1 integrin [34]			
	E-2	unknown			
	E-3	DAF [35]			
	E-4	unknown			
	E-5	Heparan sulfate [36]			
	E-6	DAF [35,37]			
	E-7	DAF [35,37,38]			
	E-9	αvβ3 integrin [39]			
	E-11	DAF [35,37], HLA Class I [40]			
	E-12	DAF [35,37]			
	E-13	DAF [38]			
	E-14	unknown			
	E-15	unknown		A1A, A1B, A2, A23, A25, A29, A30, A31, A44, A47, A49, A62	LDLR, VLDLR, LRP [41,42,43]
	E-16	unknown			
	E-17	unknown			
	E-18	unknown			
	E-19	DAF [35]			
	E-20	DAF [37]			
	E-21	DAF [37,38]			
	E-25	DAF [35]			
	E-29	DAF [38]			
	E-30	DAF [35]			
EV-C	CV-A21	ICAM-1 [44], DAF [45]	RV-B	B3, B4, B5, B6, B14, B17, B26, B27, B35, B37, B42, B48, B52, B69, B70, B72, B79, B83, B84, B86, B91, B92, B93, B97, B99, B100, B101, B102, B103, B104, B105, B106	ICAM-1 [25]
	CV-A24	unknown			
	CV-A24v	Sialic acid [46]			
	EV-C104	unknown			
	EV-C105	unknown			
	EV-C109	unknown			
	EV-C117	unknown			
	EC-C118	unknown			
	PV-3	PVR [47]			
EV-D	EV-D68	α2-6-linked sialic acids [48]	RV-C	C1, C2, C3, C4, C5, C6, C7, C8, C9, C10, C11, C12, C13, C14, C15, C16, C17, C18, C19, C20, C21, C22, C23, C24, C25, C26, C27, C28, C29, C30, C31, C32, C33, C34, C35, C36, C37, C38, C39, C40, C41, C42, C43, C44, C45, C46, C47, C48, C49, C50, C51, C52, C53, C54, C55	CDHR3 [49]

* Other co-receptors have been described including dendritic cell-specific ICAM3-grabbing non-integrin (DC-SIGN) [50], sialylated glycan [51], heparan sulfate [52], nucleolin [53], vimentin [54] and annexin II [55], but their contribution to virus entry is unclear. SCARB2: scavenger receptor class B, member 2. CAR: Coxsackievirus-adenovirus receptor. DAF: complement decay-accelerating factor, also known as CD55. ICAM-1: intercellular adhesion molecule 1, also known as CD5. LDLR: low-density lipoprotein receptor. VLDLR: very-LDLR. LRP: LDLR-related protein. CDHR3: human cadherin-related family member 3.

RV replication was shown in early experiments to be reduced at high temperatures (37 °C or 39 °C) compared to 33 °C [56]. This condition was consistent with the role of RV as an upper respiratory tract pathogen and unable to invade lower airway functioning at physiologic temperature. However, the undeniable epidemiological connection between RV infections and asthma exacerbations called into question this assumption. Since then, experimental studies have extensively reported not only effective RV replication in lower airway epithelial cells [57], but also that the difference in replication capacity at lower temperatures is minimal [58] and may vary according to the RV type [59]. RV nucleic acids have been detected also in lower airway cells by reverse transcriptase-polymerase chain reaction (RT-PCR) in bronchoscopy and bronchoalveolar lavage samples of individuals following experimental inoculation of the upper airways [60].

Other tissues have been shown to be infected by RV in addition to the nasopharynx and lower airway cells. RV RNA was detected in sinuses by RT-PCR in maxillary sinus brushings [61] and in turbinate epithelial cells in patients with chronic sinusitis [62], but also in the middle ear cavity of children with otitis media with effusion [63]. The spread to these locations is presumed to happen by local extension. Considering its theoretical restricted tropism and its sensitivity to the acid environment of the gastrointestinal tract, RV was assumed until recently to be unable to spread by viremia and to infect other organs than the respiratory tract. However, the presence of RV RNA in multiples sites, including the blood and stools has been increasingly detected in recent years and many aspects of the pathogenesis of this virus remain unclear [64,65,66,67,68]. The great number of different RV types may add an extra factor of complexity, as some are potentially more virulent than others.

#### 2.2.3. Host Response

The first line of defense against RV infection is the airway epithelium, which serves as a relatively resistant barrier against infection when undamaged and composed of well differentiated cells [69]. Early innate immune detection of RVs occurs very rapidly after infection of the epithelium and, most importantly, triggers the production and secretion of type 1 interferon (IFN), which will establish an antiviral state in the infected and surrounding cells.

At the binding step, the attachment of major group RVs to ICAM-1 activates a signaling cascade leading to the expression of chemokine genes such as C–X–C motif chemokine 1 (CXCL10) [70]. Once viral uncoating occurs, RV particles are released and activate the cell defenses. Infected cells recognize RV “pathogen-associated molecular pattern” (PAMP) by the interaction with two different families of pattern recognition receptors: toll-like receptors (TLR) and retinoic acid-inducible gene-I-like receptors (RLR), a RNA helicase family that includes retinoic acid-inducible gene-I (RIG-I), melanoma differentiation-associated gene-5 (MDA-5), and LGP-2. TLR, especially TLR-3, 7 and 8, are transmembrane receptors localized in the lumen of the endosomes that recognize viral dsRNA (for TLR-3 [71]) or ssRNA (for TLR-7 and 8 [72]) and are involved in RV genome detection [73,74,75]. In addition, TLR-2 is expressed on the cell surface and is able to detect specific molecular patterns on the viral capsid, even in the absence of replication [73]. The recognition will propagate downstream signaling and activate different transcription factors, such as interferon regulatory transcription factor (IRF) 3, IRF-7 and NF-κB, which will result in the expression of type 1 IFN and transcription of several inflammatory cytokine genes [76]. In parallel, RLR localize in the cytosol and will also recognize viral genomes. MDA-5 binds to dsRNA generated as an intermediate of replication and therefore has an important role in the anti-RV response [77], whereas RIG-I binds to the 5′ triphosphate motif and has a more controversial role in the recognition of picornaviruses. Indeed, picornaviruses do not exhibit the 5′triphosphate motif, but instead the 5′end of their genome is covalently linked to the viral genome-linked protein (VPg) and their recognition is mediated principally through MDA-5 [77,78]. Upon ligand recognition, RLR activate the mitochondrial adaptor molecule MAVS (also named VISA, Cardif and IPS-1), which will activate the IFN induction pathway using the same intermediates.

RV infection triggers the release of a variety of antiviral factors and cytokines, including bradykinin, IL-1β, TNFα, IL-6 and IL-8, activating and attracting granulocytes, dendritic cells, and monocytes at the site of infection [79]. An antibody response to RV infection also occurs with the development of serotype-specific neutralizing serum antibodies (IgG) and secretory antibodies (IgA) in the airways, detectable usually after one or two weeks after inoculation and maintained for at least one year [80]. As RV infections are generally short-lived, these neutralizing antibodies appear after viral clearance, but have an essential role in protecting from reinfection from the same type of virus [81]. This humoral response seems to be serotype-specific with only little antibody cross-reacting, which represents a difficult challenge for vaccine development. However, this pivotal question remains controversial [82,83].

#### 2.2.4. Clinical Syndromes and Epidemiology

RV was found to be the etiology of one-half to two-thirds of common colds [1] and is therefore regarded as the most common human infectious agent worldwide. Children are considered as the major reservoir for RVs and experience up to eight to 12 infections per year, whereas adults are infected two to three times per year [84]. The average incubation period is two days [85] after nasal inoculation with a symptom duration of seven to 10 days [86]. Infections occur all year round, but two peaks of infection are classically reported, the first between April and May and the second between September and October [87,88]. Interestingly, a recent study showed that RV-Cs demonstrate a different trend, with a peak of infection during winter months [89]. The only known host of RV is human, even if primates might also be susceptible to asymptomatic infection [90,91]. In immunocompetent individuals, the virus is usually strictly restricted to the upper respiratory airways and typically induces nasal congestion and rhinorrhea, cough, sneezing, sore throat and malaise, with a spontaneous resolution within one to two weeks [92]. However, RVs can also cause a wide range of respiratory illnesses, ranging from asymptomatic infection to bronchitis and wheezing, bronchiolitis, or pneumonia. Rare cases of extra-pulmonary illnesses related to RV have been recently described, including gastroenteritis [65,93] and pericarditis cases [94].

#### 2.2.5. Animal Models

To elucidate the pathogenesis of RV infection in human, a reliable animal model would be essential. However the host range of RV is very limited, as they are able to replicate only in cells of primate origin. These limitations are believed to result in part from cellular receptor incompatibility (which is the case for major group RV types but not for minor group RVs) and also from post-entry intracellular block to replication [95,96,97,98]. Mice adapted viral strains or transgenic mice expressing the human cellular receptor ICAM-1 have been successfully developed, however these models inadequately mimic an infection in the natural host [99,100,101]. The lack of adequate animal model to study the pathogenesis of RV has been partially overcome by the development of *in vitro* reconstituted differentiated human airway epithelia. These tissues exhibit *in vivo* morphological and growth characteristics of the respiratory epithelium and allow a thorough analysis of some aspects of the pathogenesis of RV (see Section 3.3.1).

## 3. Update on Latest Findings on RVs and Respiratory EVs

### 3.1. RV and EV Classification: Current Status

**Figure 2:**
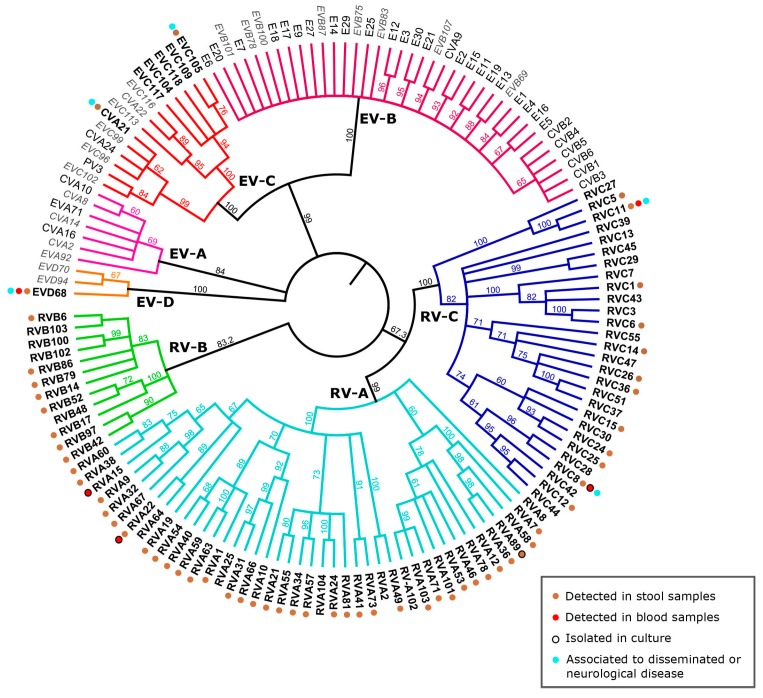
Phylogeny of the different human enterovirus species with emphasis on respiratory enteroviruses and associated clinical features. The VP1 nucleotide region of selected representatives of RV-A to -C and EV-A to -D species including all non-RV EVs associated with respiratory disease (Table 1) were included in the phylogenetic analysis with simian sapelovirus 1 (SSV-1) as the outgroup (see Table S1 for Genbank IDs). The tree was computed as previously described [6]. The consensus tree resulting from PhyML analysis is shown as cladogram. Names of viruses detected predominantly or exclusively in respiratory samples are in bold, names of viruses detected occasionally in respiratory samples are in black while names of non respiratory EVs are in light grey and italic. Clinical features associated with viruses predominantly detected in respiratory samples are color-coded. The references for each unusual associated symptom or detection site are available in Table S2.

The EV genus belongs to the *Picornaviridae* family, which is composed of some of the simplest RNA viruses containing very limited genetic material. Despite this genomic size constraint, the *Picornaviridae* family displays a great variability between its different members and a very large number of types can be distinguished. Historically, EVs and RVs were classified into separate genera, but due to their closely related genome organization and structure, they were merged into a single genus named EV. This genus is divided into 12 species, based on the genetic homology and similarity of pathophysiology [102]. Seven of these species are composed of human pathogens: three RV species (RV-A, RV-B, RV-C) and four EV species (EV-A, EV-B, EV-C, and EV-D) (Figure 2). Formerly named human rhinovirus (HRV) and human enterovirus (HEV) species, the International Committee on Taxonomy of Viruses (ICTV) decided in February 2013 to abandon host names and renamed these species simply as Rhinovirus-A, B and C and Enterovirus-A, B, C and D [102,103]. Between 1956 and 1987, 101 different serotypes of RV were established based on serological cross-neutralization assays in cell culture [104] and were divided into species RV-A and RV-B [105]. Similarly, 66 human EV serotypes were defined until 1999 by serum neutralization. This method of classification was insensitive, time-consuming, and labor intensive and was restricted by the limited supply of standardized antisera [106] and the inability to isolate some EV or RV types in cell culture. A molecular typing system was developed in 1999 for EV, which relies on the sequencing of an amplicon targeting a variable part of VP1. To date, the accepted threshold for type assignment is >25% divergence in the VP1 coding region [107].

The serological method of classification based on the isolation of viruses in culture cells prevented the detection of an entire species of RV, now known as RV-C, which are uncultivable on standard cell cultures. The arrival of molecular detection techniques allowed the discovery in 2006 of this new RV species, composed of 55 different types to date [102]. Inspired by the non-RV EV classification system, a genetically-based classification for RV was proposed by Simmonds *et al.* [108], also determined by the divergence of the VP1 nucleotide sequence. At the same time, the name “serotype” was changed to “genotype” or simply “type”. A threshold of 13% divergence on VP1 nucleotide sequences was proposed for type assignment and is still currently accepted. This classification system was extended a few years later for species A and B and it was established that a RV type should have at least 13% (for RV-A), 12% (RV-B), or 13% (RV-C) nucleotide divergence from all other RV types [109].

In the EV genus, members of a same species must share >60% amino acid identity in P1 (capsid proteins), >70% in 2C + 3CD (non-structural proteins) and in the polyprotein sequence, as well as sharing a limited natural host range, a limited range of cell receptor, a genome composition (G + C) that varies of less than 2.5%, and a considerable degree of compatibility in proteolytic processing, replication, encapsidation and genetic recombination.

### 3.2. Mechanisms of RV Evolution and Adaptation

The vast genetic diversity of non-RV EV and RV types is an important characteristic of these viruses and explains a substantial part of the variety of their associated clinical syndromes. Two driving forces for this diversity coexist: the high error-rate of the viral RNA-dependent RNA-polymerase (RdRp), and the occurrence of recombination events.

In general, RNA viruses display a great genetic diversity, mostly arising from the high mutation rate caused by the error-prone nature of their RdRp, which misincorporates nucleotides at a frequency of 10^−3^ to 10^−5^ per nucleotide site [110]. For EV, this mutation frequency is believed to give rise to approximately one mutation introduced per genome replication. The resulting collection of the multitude of related, but non-identical viral variants, is referred to as quasispecies. This high level of genetic diversity results in a great variety of amino acid sequences of the capsid region, which can explain the large number of different recognized RV types. *In vivo*, the mutation frequency in the open reading frame of a RV-A39 during a 5-day course of infection in healthy, experimentally-inoculated volunteers could be estimated at 3.4 × 10^−4^ changes/nt, equivalent to 6.83 × 10^−5^ changes/nt/day [111]. Another study of RV genome evolution in immunocompromised patients revealed a RV mutation frequency of the same order of magnitude, *i.e.*, between 7.27 × 10^−6^ to 3.88 × 10^−5^ mutation/nt/day [112].

By contrast, recombination events occur very frequently, particularly within non-RV EVs, and participate extensively in the genetic diversity of these viruses. Two different molecular mechanisms underlying RNA virus recombination are currently believed to exist: (1) a classic replicative model with template-switching of the viral polymerase occurring during replication and giving rise to homologous recombinants; and (2) a non-replicative model where the cut and rejoining of different viral RNA fragments occur by an as yet unknown mechanism and create non-homologous recombinants with sequence duplications at gene boundaries [113,114,115,116,117,118,119]. The homologous replicative recombination may be of greater importance under natural conditions, but the relative importance of both mechanisms remain unclear. In non-RV EV, recombination has been extensively studied and documented and is an undeniable force of evolution of these viruses, occurring at high frequency mostly in non-structural regions [120,121,122]. Interestingly, recombination in RV seems to be surprisingly rare and is probably mostly limited to ancient events. Based on sequence analysis, it was speculated that recombination could be at the origin of RV-B species, which would have been generated by recombination between RV-A and non-RV EV ancestors [123]. Interspecies recombination between RV-A and RV-C in the 5′UTR and 2A sequences have been identified and are certainly the result of an ancient evolutionary event [124]. On the other hand, contemporary recombination events among RV circulating strains are believed to occur mainly between the same species and thus would give rise to recombinants highly related to the parental strains. Contemporary intraspecies recombinations within the coding region have been documented for RV-A [6], but not for RV-B and -C [6,109]. Experimental investigations of RV recombination have attempted to elucidate this phenomenon by studying the genetic exchangeability between different RV strains. By artificially exchanging some sequences, it was possible to show that intra- and interspecies RV/RV and RV/non-RV EV exchanges in the 5′UTR could give rise to fully viable viral chimeras [125]. These viruses could be easily propagated in cell cultures, but were not able to outcompete the better-adapted parental strain. By contrast, interchangeability potential in the polyprotein coding regions seems to be reduced. When engineering artificial recombinants where the capsid together with the 2A-encoding region is exchanged, only intraspecies exchanges can give rise to viable viruses. Similarly, non-replicative recombination occurred only between genomes belonging to the same RV-A species [119]. Again, fitness of the different recombinants was reduced compared to the parental virus, thus indicating that this kind of event would not easily happen in nature [119].

The high heterogeneity in the coding sequence of RV was speculated to be an important obstacle to the emergence of viable recombinants compared to non-RV EV and may be a partial explanation of the lower frequency of these events in nature [124]. Another possible explanation may lie in the great difference between RV and non-RV EV pathogenesis. Some characteristics of RV infection could be a limitation to spontaneous recombination *in vivo*, either in terms of restricted tropism or short duration of infection. However, this is only speculative and RV recombination mechanisms and limitations are still far from being fully understood.

### 3.3. Recent Advances in Knowledge of the In Vitro and in Vivo Pathogenesis of RV and Respiratory EV

#### 3.3.1. RV-C: Getting to Know the Newcomers

The arrival of new molecular diagnostic tools allowed the discovery in 2006 of a new species of RV (RV-C) [126,127,128,129,130,131,132,133] that had remained undetectable until then due to the incapacity of these viruses to grow in standard cell lines. This species is not composed of emergent strains and has been circulating in humans for at least 250 years [134]. Since its discovery, RV-C is reported to have a high prevalence and in that respect resembles more RV-A than the less prevalent RV-B [135,136]. Epidemiological data revealed that its seasonality seem to differ from the other RV species, with a peak of infection during the winter months [89]. Clinical manifestations associated with RV-C seem to be more severe in children [135] and also more often disseminated [6,94,126,137,138,139,140,141]. Of note, a case study of a systemic RV-C type 8 infection causing a fatal acute respiratory illness in a young child was reported recently, with isolation of the virus from blood and detection of nucleic acids in different body sites (lungs, blood, gastrointestinal tract, and cerebrospinal fluid) [64] (Figure 2). However, the association between RV-C and more severe illness is controversial and other studies showed no difference in clinical presentation and severity among different RV species infections, particularly between RV-A and -C infections [112,129,135,142,143,144,145]. Recent data have suggested that there may be age differences in the prevalence and severity associated with RV species. RV-A is more frequent in adults, while RV-C is more frequent among children [146] and appears to be more severe in this population, with a significantly higher rate of lower respiratory tract infections than observed in adults [147]. Finally, a recent prospective study evaluating the circulation of different types of RV among young children reported that RV infection is extremely frequent in this population, but remained asymptomatic in 64% [148,149]. Defining the exact etiological role of RV in respiratory disease remains difficult, including the association of a particular species or even type with specific clinical findings.

The inability of RV-C to propagate in standard immortalized cell lines has hampered research progress related to the biological properties of this new group of viruses. Bioinformatic comparisons of sequences suggested a different receptor use than RV-A and -B, which may explain this distinctive growth feature, even if another receptor-independent limitation could not be ruled out [150]. A first successful amplification of RV-C15 clinical specimens was reported in sinus mucosal organ cultures, obtained as a byproduct of human sinus surgery [150]. RV-C15 and -C11 generated from infectious cDNA clones could be amplified in fully differentiated human AECs [151], similar to RV-C15 and -C41 clinical specimens in differentiated sinus epithelial cells cultured under air-liquid interface (ALI) conditions. Another ALI culture system using 3D human upper airway epithelia reconstituted *in vitro* allowed the growth of five different types of RV-C and the study of biological properties of these viruses [59]. A comparative study of RV-A, -B, and -C replication and inflammatory response induction was performed in an ALI culture system and showed that RV-B subtypes exhibit slower and lower replication, but also induce lower cytotoxicity and cytokine production [152]. These results are consistent with clinical observations and epidemiological data indicating that RV-B types cause less severe illness than RV-A or -C types [135]. With these models it could also be confirmed that similar to RV-A and -B, RV-C is acid-sensitive, although temperature sensitivity seemed to differ from one type to another. Some RV-C types can grow efficiently in higher temperature conditions, which could be an explanation for the apparent greater propensity of some strains to cause lower airway syndromes [59].

The next major step forward in the understanding of RV-C pathogenesis was the very recent discovery of the cell receptor used by these viruses. By comparing genome-wide gene expression analysis between cells susceptible *versus* not susceptible to RV-C infection, Bochkov *et al.* [49] found 400 genes expressed exclusively in RV-C permissive cells. They selected 12 that are common genes encoding membrane-bound proteins and then functionally validated them by transfecting HeLa cells with plasmid DNAs encoding these genes. A reporter virus (RV-C15-GFP) was inoculated in these different cells and only cells transfected with cadherin-related family member 3 (CDHR3) became permissive to RV-C infection. Replication of other RV-C strains in stably expressing CDHR3 cells that were previously insensible to RV-C infection was also demonstrated, suggesting that CDHR3 could be the functional receptor for RV-C [49]. CDHR3 is highly expressed in airway epithelium and as a cadherin family member is supposed to be involved in cell adhesion, cell-cell interaction, and epithelium polarity and differentiation. Interestingly, a single nucleotide polymorphism (SNP) in this gene was previously associated by genetic analysis with severe asthma exacerbation in children [153]. When tested *in vitro*, this SNP allowed increased RV-C binding and replication, thus confirming the link between RV infection and severe asthma. This finding represents a key step in the study of RV-C by providing a useful insight into the biological properties of these viruses and will most likely be determinant in the discovery of new RV-C inhibitors.

#### 3.3.2. Recent Re-Emergence of EV-D68 and Other Respiratory EVs

EV-D68 is a member of the small EV-D species and was first isolated in 1962 in California, USA, in respiratory samples of four children with respiratory disease [154]. Due to its biological properties, such as the typical acid lability of RV and an optimal growth temperature of 33 °C, EV-D68 is of particular interest because it shares characteristics of both RV and EV [103,155]. Isolated from respiratory samples, some strains of EV-D68 were independently classified initially in the RV genus as RV87. However, following genetic and antigenic studies, it was determined that they were similar to EV-D68 strains and all RV87 strains have now been reclassified as EV-D68 type [103]. Rarely observed until the late 2000s, a few clusters of EV-D68 cases were progressively reported in different parts of the world during the last decade and associated with mild to severe respiratory illness [156,157,158,159]. During autumn 2014, the USA experienced the largest outbreak of EV-D68 with an unprecedented level of circulation nationwide, especially in the pediatric population. A total of 1153 individuals in 49 states and the District of Columbia tested positive for this virus, mostly children, many with a previous history of wheezing or asthma [160].

This rapid increase in reported cases over the last few years was first believed to be caused by the improvement of detection techniques and to the previous misidentification of EV-D68 as a RV leading to an underestimated prevalence. However, retrospective tests confirmed this real increase in prevalence [158,161]. Phylogenetic analysis of recently detected EV-D68 strains revealed an increased diversity in VP1 sequences. These strains cluster in three different genetic lineages, which are clearly distinguishable from the prototype strains [158,161]. Some amino acids changes (mostly substitutions but also one deletion) in the capsid encoding genes, predominantly in VP1, define these different lineages. The regions involved are located in the putative immunogenic BC and DE loops. This finding is consistent with the demonstration of Imamura *et al.* [48] that these emergent strains have highly different antigenic properties, which could have impacted greatly on the transmission dynamics of the virus and may explain the epidemiological change. Other surprising genetic variations that are described consist in the presence of deletions in the 3′end of the 5′UTR, which is usually considered to be the most conserved region among EVs [48]. These deletions occur in a spacer region, between the IRES and the ORF of VP4. As the function of this spacer region is unclear, it is difficult to assess if these variations confer an advantage for these emergent strains, and if these mutations can be considered as potential genetic markers of virulence.

Concurrently with its respiratory tropism, EV-D68 infections have been increasingly associated with neurologic disease [13,162,163], including cases of acute flaccid myelitis, thus suggesting a link between EV-D68 and this type of complications [164,165,166]. In one case, EV-D68 neurotropism was confirmed by detection of the virus in the cerebrospinal fluid and brain at autopsy of a 5-year-old boy with fulminant encephalitis [162]. EV-D68 was also detected in a blood sample of a child with acute flaccid paralysis (AFP) [167]. However, although many epidemiological and clinical factors suggest this association [167], the direct causality link between EV-D68 and neurological involvement has never been strictly demonstrated as reports of association occurred in a period of high EV-D68 incidence and could have been coincidental. Further investigations are thus still needed to prove this association.

The receptor binding molecules utilized by EV-D68 were found to be sialic acids (SA), similar to many other viruses, with a higher affinity for α-2-6-linked SA than α-2-3-linked SA [48]. These sialylated glycans are extensively expressed on the outer cell membranes of the human airway tract [168]. Two other EVs were found to have an affinity for sialic acids, EV-D70 (a close relative of EV-D68) [169] and the coxsackievirus A24 variant (CVA24v, member of EV-C) [46,170,171]. These viruses are causative agents of acute hemorrhagic conjunctivitis, but can also cause symptoms in the upper respiratory tract and neurological impairment such as acute flaccid paralysis, and are considered to have a pandemic potential [46]. Even if the pathogenesis of these three viruses varies significantly, this common receptor shared by potentially highly contagious viruses suggests a common mechanism that needs to be further investigated.

Other non-RV EVs from species C have been recently discovered and show a predominant respiratory tropism (Figure 2). EV-C104 [6], EV-C105 [7,172], EV-C109 [8], EV-C117 [9], and EV-C118 [10] have been discovered during the last six years and form a distinct clade within the EV-C species. These viruses show a worldwide distribution [8,173,174,175] and cause diseases of varying severity ranging from asymptomatic or mild respiratory infections to complicated diseases, such as pneumonia [10,175,176]. Of note, there is also a report of EV-C105 detection in the rectal swab of a fatal acute flaccid paralysis patient [7,172]. If confirmed, such observations would suggest that some respiratory EVs from species C may infect the gut and reach the central nervous system. Rarity of detection, combined with difficulties in propagating these viruses in culture, limits our ability to investigate their biology and genetic diversity and their host cell receptors are not known at the present time [8,9,176,177]. Another EV-C, coxsackievirus-A21 (CVA21) is associated with mild respiratory illness [178]. Interestingly, it utilizes ICAM-1 as receptor, the same molecule used by the major receptor group of RV [45,179] (Table 2). This virus revealed to be able to invade the central nervous system by retrograde axonal transport and caused poliomyelitis in ICAM-1 transgenic mice [180] In humans, CVA21 was detected in stool samples of patients with AFP [181].

Of note, EV-A71, which came up in the last decade as a major cause of Hand-Foot-Mouth-Disease and viral encephalitis in the Asia-Pacific region [182,183], is also a common agent of respiratory disease. Associated to pharyngitis, bronchiolitis, croup and pneumonia, EV-A71 infections mostly affect young children [184,185,186]. In addition, cases of acute pulmonary edema have been associated to severe EV-A71 encephalitis, but these are believed to result from the destruction of medullary vasomotor and respiratory centers even if the exact pathogenesis is not completely understood [187].

In summary, emerging or re-emerging respiratory EVs have been highlighted in recent years and their circulation should be closely monitored, particularly EV-D68 and EV from species C exhibiting potential neurotropic features.

### 3.4. Optimal Growth Temperature of RV: A Possible Link with the Interferon Response

RVs are known to optimally grow at cooler temperatures than non-RV EVs [56]. Until recently, this was assumed to partially explain their restricted tropism to the nasal cavity. The mechanism of this growth pattern remains unknown, despite years of research. The viral replication machinery was first believed to be involved. However, studies of cell entry, uncoating, or polymerase activity could not find a virus-intrinsic reason for this temperature-dependence [188,189]. Foxman *et al.* [190] investigated the possibility that this characteristic of RVs may depend on the capacity of defense of the victim, instead of a weakness of the assailant. The cellular innate immune response repressing RV infection could be more effective at higher temperatures, thus inhibiting an efficient RV replication and infection. By contrast, cells would be more vulnerable to RV infection at lower temperatures.

By using a mouse model system and a mouse-adapted variant of RV-1B (minor receptor group), Foxman *et al.* [190] examined host-virus interactions and, more precisely, the IFN response to infection at 33 °C compared to 37 °C in primary AECs. As expected, viral replication was less efficient at non-permissive temperatures and an earlier plateau in viral titer was reached at this temperature. The unexpected finding was that this earlier plateau could be correlated to an increased induction of IFN (protein and mRNA), as well as IFN-stimulated genes (ISGs) at this temperature. Using AECs from knockout mice lacking innate immune signaling molecules, it could be demonstrated that the recognition pathway involved in this temperature-dependent IFN induction is the RLR receptor pathway, and that the PAMP eliciting this response would probably be viral replication by recognition of dsRNA (a replication intermediate). When investigating further the mechanism of this increased induction of the IFN pathway, they observed that the levels of IFN secretion were higher at 37 °C than at 33 °C at fixed concentrations of RLR ligands (by using the synthetic poly I:C ligand), thus revealing an enhanced RLR function at a higher temperature. The direct function of RLR seems to be improved also, as demonstrated by the better ability of receptors to catalyze ATP hydrolysis at 37 °C than 33 °C. Finally, the authors compared the viral replication in AECs deficient in the RLR detection pathway and infected at 37 °C to wild-type AECs infected at 33 °C. It was demonstrated that growth was almost similar, proving a significant contribution of innate immunity to the temperature-dependent growth of RVs.

By showing that the modification of temperature impacts the immune response to infection rather than, or in addition to a virus-intrinsic property, Foxman *et al.* [190] answered a fundamental question about RV pathogenesis. However, more generally, this opened the door to a new understanding of innate immunity functioning. The idea that RV infection, particularly URTI, is linked to exposure to cold air is a widespread and long-standing popular belief that has been extensively studied over the last centuries without finding a direct pathophysiological effect. To some extent, the work of Foxman *et al.* [190] is the first demonstration of how temperature can directly impact virus-host interaction and weakens the innate immune response to infection. Testing other RV strains, but also other respiratory viruses, could bring a better understanding of this function and perhaps allow a generalization of this immune mechanism in the host antiviral response. A limitation of this study is the use of a mouse model system and mouse-adapted virus and it would be interesting to confirm these results in a human model, such as a three-dimensional human airway epithelia reconstituted *in vitro* [59].

## 4. Conclusions

RVs and respiratory EVs have been extensively studied during the past years and substantial progress has been made towards a better understanding of their biology. The development of new molecular tools has allowed the discovery of an entire species of RV, RV-C, which had remained undetected due to its inability to grow in standard cell culture. Since then, viruses from this species have been increasingly detected and their clinical importance is now undeniable. A significant advance in the understanding of their specific biological properties was the recent identification of the RV-C cellular receptor, which gives a useful insight into the early mechanism of infection of these viruses. Many host cell receptors of respiratory EVs remain unknown, especially for non-RV EVs. These cell receptors are believed to be important determinants of pathogenesis and cell tropism and it would be of great relevance to identify these molecules. Finding RV-C cell receptor, in addition to other key information on these viruses was made possible thanks to the development of new functional culture systems, which allowed the growth and characterization of difficult to study respiratory pathogens. *In vitro* reconstituted 3D human airway epithelial tissues or other types of differentiated epithelial cells cultured under air-liquid interface conditions represent a useful tool to study RV infection and suggest interesting prospects in improving our understanding of the biology of these viruses.

Genetic analyses of variants found in clinical screenings have led to the discovery of many novel RV and EV strains during the last few years. A great genetic diversity driven by mutations and recombination characterizes EV and RV and leads to an impressive number of different types and variable clinical presentations. Exploring the driving forces behind this evolution may help to understand the evolutionary pattern of these viruses and to anticipate the emergence or re-emergence of better-adapted strains. One of these reemerging strains that has been particularly highlighted during the past year is EV-D68, which is believed to be a non-RV EV with an probable exclusive respiratory tropism. Rarely detected until recently, this virus was able to cause an outbreak of respiratory disease in the pediatric population in North America. In addition a possible role of EV-D68 infection in acute flaccid myelitis cases has been suggested, albeit not proven. This is a concrete example of how a more detailed understanding of EV genetic determinants would help to appreciate the impact on viral properties of the emergence of new mutations and lineages within a particular subtype.

Further studies are needed to improve our understanding of the pathogenesis of these highly prevalent viruses. This is particularly essential in the light of their significance for public health and the considerable associated clinical morbidity. Direct and indirect costs resulting from RV and non-RV EV infections place a heavy financial burden on healthcare systems worldwide. Total costs associated with RV infection in the USA were estimated at approximately US$40 billion per year, which is greater than many other conditions, such as hypertension, asthma, or chronic obstructive pulmonary disease [2]. Numerous EV inhibitors have been shown to be promising, but these are currently still under study and are not yet commercialized. All strategies regarding the development of vaccines against non-RV EV (except poliovirus) and RV have failed so far, primarily because of the lack of cross-protection between the different subtypes. Improving our knowledge of RV evolution and diversity is crucial to have a reasonable hope of success in finding new antiviral targets.

## Supplementary Materials

The following are available online at http://www.mdpi.com/1999-4915/8/1/16/s1, Table S1: Genbank IDs of selected representatives of RV-A to -C , EV-A to -D and Simian Sapelovirus species that were included in the phylogenetic analysis in Figure 2, Table S2: References citing unusual symptoms or detection sites of respiratory EVs as listed in Figure 2.
