# Supplementary material for: Rhinoviruses and Respiratory Enteroviruses: Not as Simple as ABC
Source: Viruses. 2016 Jan 11;8(1):16. doi: 10.3390/v8010016 (PMC4728576; doi:10.3390/v8010016)
Supplement: Supplementary file 1 [file viruses-08-00016-s001.pdf]

Supplementary Material

Table S1. Genbank IDs of selected representatives of RV-A to -C , EV-A to -D and Simian Sapelovirus species that were included in the phylogenetic analysis in Figure 2.

Non-Rhinovirus Enterovirus Species									
EV-A		EV-B				EV-C		EV-D	
Type	Genbank ID	Type	Genbank ID	Type	Genbank ID	Type	Genbank ID	Type	Genbank ID
CV-A2	AY421760	CV-A9	D00627	E-17	AY302543	PV-1	V01148	D68	AY426531
CV-A8	AY421766	CV-B1	M16560	E-18	AF317694	PV-3	KJ170670	D70	DQ201177
CV-A10	AY421767	CV-B2	AF081485	E-19	AY302544	CV-A17	AF499639	D94	DQ916376
CV-A12	AY421768	CV-B3	M88483	E-20	AY302546	CV-A19	AF499641	D111	JF416935
CV-A14	AY421769	CV-B4	S76772	E-21	AY302547	CV-A22	AF499643	D120	KF040080
CV-A16	AF177911	CV-B5	AF114383	E-25	AY302549	CV-A24	D90457	–	–
EV-A71	U22522	CV-B6	KT380953	E-27	AY302551	EV-C96	EF015886	–	–
EV-A89	AY697459	E-1	AF029859	E-29	AY302552	EV-C99	EF555644	–	–
EV-A91	AY697461	E-2	AY302545	E-30	AF162711	EV-C102	EF555645	–	–
EV-A92	EF667344	E-3	AY302553	EV-B69	AY302560	EV-C104	EU840733	–	–
EV-A119	KC787153	E-4	AY302557	EV-B74	AY556057	EV-C105	JF838290	–	–
–	–	E-5	AF083069	EV-B75	AY556070	EV-C109	GQ865517	–	–
–	–	E-6	AY302558	EV-B78	AY208120	EV-C113	KC344833	–	–
–	–	E-7	AY302559	EV-B83	AY843301	EV-C116	JX514942	–	–
–	–	E-9	AF524866	EV-B87	AY843305	EV-C117	JX262382	–	–
–	–	E-11	X80059	EV-B98	AB426608	EV-C118	JX393301	–	–
–	–	E-12	X79047	EV-B100	DQ902713	–	–	–	–
–	–	E-13	AY302539	EV-B101	AY843308	–	–	–	–
–	–	E-14	AY302540	EV-B106	KF990476	–	–	–	–
–	–	E-15	AY302541	EV-B107	AB426609	–	–	–	–
–	–	E-16	AY302542	EV-B111	KF312882	–	–	–	–

Table 1. *Cont.*

**Rhinovirus Species**										
**RV-A**		**RV-B**				**RV-C**				
Type	Genbank ID	Type	Genbank ID	Type	Genbank ID	Type	Genbank ID	Type	Genbank ID	
A1	FJ445111	A55	DQ473511	RV-B6	DQ473486	C1	EF077279	C45	JN837686	
A2	X02316	A57	FJ445141	RV-B14	L05355	C3	JN798567	C47	JF519760	
A7	FJ445176	A58	FJ445142	RV-B17	EF173420	C4	EF582385	C48	JF519762	
A8	FJ445113	A59	DQ473500	RV-B42	FJ445130	C5	EF582386	C51	JX291115	
A9	FJ445177	A60	FJ445143	RV-B48	DQ473488	C6	JN815245	C55	529T	
A10	DQ473498	A61	FJ445144	RV-B52	FJ445188	C7	DQ875932	–	–	
A12	EF173415	A63	FJ445146	RV-B70	DQ473489	C8	GQ223227	–	–	
A15	DQ473493	A64	FJ445181	RV-B79	FJ445155	C11	EU840952	–	–	
A16	L24917	A65	FJ445147	RV-B86	FJ445164	C12	HM236958	–	–	
A19	FJ445119	A66	FJ445148	RV-B92	FJ445169	C13	HM236908	–	–	
A21	FJ445121	A67	FJ445149	RV-B93	EF173425	C14	HM236911	–	–	
A22	FJ445122	A71	FJ445152	RV-B97	FJ445172	C15	GU219984	–	–	
A24	FJ445190	A73	DQ473492	RV-B99	FJ445174	C24	HM236939	–	–	
A25	FJ445123	A74	DQ473494	RV-B100	HQ123444	C25	JN837685	–	–	
A29	FJ445125	A76	FJ445182	RV-B101	JF781500	C26	HM236904	–	–	
A31	FJ445126	A78	FJ445183	RV-B102	JX074053	C27	HM236906	–	–	
A32	FJ445127	A81	FJ445157	RV-B103	JQ994497	C28	JN798569	–	–	
A34	FJ445189	A82	FJ445160	RV-B104	FJ445137	C29	HM236949	–	–	
A36	DQ473505	A89	FJ445184	–	–	C30	HM236968	–	–	
A38	FJ445180	A100	FJ445175	–	–	C31	HM236964	–	–	
A39	AY751783	A101	GQ415051	–	–	C36	JN541267	–	–	
A40	FJ445129	A102	EF155421	–	–	C37	JF416321	–	–	
A41	DQ473491	A103	JF965515	–	–	C39	JN205461	–	–	
A46	DQ473506	A104	JX193797	–	–	C42	JQ994500	–	–	
A49	DQ473496	A105	JN614995	–	–	C43	JF416307	–	–	
A53	DQ473507	A106	JX025555	–	–	C44	JF416310	–	–	

**Sapelovirus Species**	
**SSV-B**	
Type	Genbank ID
SSV1	AY064708

Table S2. References citing unusual symptoms or detection sites of respiratory EVs as listed in Figure 2.

Clinical features associated with EVs or RVs	References
**RV-A Detected in Stool Samples**	
A1, A7, A9, A10, A12, A19, A21, A22, A24, A31, A32, A34, A36, A38, A40, A41, A46, A49, A53, A55, A54, A57, A58, A59, A60, A61, A63, A66, A67, A71, A73, A78, A81, A89, A101, A103	[1,2]
**RV-A Isolated in Culture from Stool Sample**	
A89	[2]
**RV-A Detected and Isolated in Culture from Blood Samples**	
A15, A22	[3]
**RV-B Detected in Stool Samples**	
B6, B14, B17, B42, B52, B79, B86, B97	[1,2]
**RV-C Detected in Stool Samples**	
C1, C5, C6, C12, C14, C15, C24, C25, C26, C36, C37, C39, C42, C43, C45	[1,2]
**RV-C Detected in Blood Samples**	
C8, C11	[4,5]
**RV-C Isolated in Culture from Blood Sample**	
C8	[4]
**RV-C Associated to Disseminated or Neurological Disease**	
C8, C11	[4,5]
**EV-C detected in stool samples**	
C105, CVA21	[6,7]
**EV-C Associated to Disseminated or Neurological Disease**	
C105, CVA21	[6,7]
**EV-D Detected in Stool Samples**	
D68	[8]
**EV-D Detected in Blood Samples**	
D68	[8]
**EV-D Associated to Disseminated or Neurological Disease**	
D68	[8]

References

1. Harvala, H.; McIntyre, C.L.; McLeish, N.J.; Kondracka, J.; Palmer, J.; Molyneaux, P.; Gunson, R.; Bennett, S.; Templeton, K.; Simmonds, P. High detection frequency and viral loads of human rhinovirus species a to C in fecal samples; diagnostic and clinical implications. *J. Med. Virol.* **2012**, *84*, 536–542.
2. Honkanen, H.; Oikarinen, S.; Peltonen, P.; Simell, O.; Ilonen, J.; Veijola, R.; Knip, M.; Hyoty, H. Human rhinoviruses including group c are common in stool samples of young finnish children. *J. Clin. Virol.* **2013**, *56*, 250–254.
3. Urquhart, G.E.; Stott, E.J. Rhinoviraemia. *Br. Med. J.* **1970**, *4*, 28–30.
4. Lupo, J.; Schuffenecker, I.; Morel-Baccard, C.; Bardet, J.; Payen, V.; Kaiser, L.; Constant, S.; Lobrinus, J.A.; Lin-Marq, N.; Lina, B.; *et al.* Disseminated rhinovirus C8 infection with infectious virus in blood and fatal outcome in a child with repeated episodes of bronchiolitis. *J. Clin. Microbiol.* **2015**, *53*, 1775–1777.
5. Tapparel, C.; L'Huillier, A.G.; Rougemont, A.L.; Beghetti, M.; Barazzone-Argiroffo, C.; Kaiser, L. Pneumonia and pericarditis in a child with hrv-c infection: A case report. *J. Clin. Virol.* **2009**, *45*, 157–160.
6. Lukashev, A.N.; Drexler, J.F.; Kotova, V.O.; Amjaga, E.N.; Reznik, V.I.; Gmyl, A.P.; Grard, G.; Taty Taty, R.; Trotsenko, O.E.; Leroy, E.M.; *et al.* Novel serotypes 105 and 116 are members of distinct subgroups of human enterovirus C. *J. Gen. Virol.* **2012**, *93*, 2357–2362.
7. Wang, S.; Xu, M.; Lin, X.; Liu, Y.; Xiong, P.; Wang, L.; Xu, A.; Tao, Z.; Zhang, D. Molecular characterization of coxsackievirus A21 in shandong, China. *Arch. Virol.* **2015**, doi:10.1007/s00705-015-2669-7.
8. Greninger, A.L.; Naccache, S.N.; Messacar, K.; Clayton, A.; Yu, G.; Somasekar, S.; Federman, S.; Stryke, D.; Anderson, C.; Yagi, S.; *et al.* A novel outbreak enterovirus d68 strain associated with acute flaccid myelitis cases in the USA (2012-14): A retrospective cohort study. *Lancet Infect. Dis.* **2015**, *15*, 671–682.

© 2016 by the authors; licensee MDPI, Basel, Switzerland. This article is an open access article distributed under the terms and conditions of the Creative Commons by Attribution (CC-BY) license (http://creativecommons.org/licenses/by/4.0/).
